# Robust Increase in IQCK Protein Expression in Mouse Models of Alzheimer's Disease and iPSC‐Derived Neurons

**DOI:** 10.1111/jcmm.70686

**Published:** 2025-07-02

**Authors:** Juliet Akkaoui, Dinesh Devadoss, Hongjie Wang, Alexandru Movila, Madepalli K. Lakshmana

**Affiliations:** ^1^ Department of Cellular and Molecular Medicine Herbert Wertheim College of Medicine, Florida International University Miami Florida USA; ^2^ Department of Chemistry and Biochemistry, Center for Molecular Biology and Biotechnology, Institute for Human Health and Disease Intervention (I‐HEALTH) Florida Atlantic University Jupiter Florida USA; ^3^ Department of Biomedical and Applied Sciences Indiana University School of Dentistry Indianapolis Indiana USA; ^4^ Indiana Center for Musculoskeletal Health Indiana University School of Medicine Indianapolis Indiana USA; ^5^ Richard L. Roudebush VA Medical Center Indianapolis Indiana USA

**Keywords:** 3xTg mice, Aging, Alzheimer's disease, APΔE9 mice, Brain regions, Dendrites, iPSC neurons, IQCK

## Abstract

Emerging studies indicate that the IQ‐motif‐containing protein K (IQCK) is a novel risk factor for Alzheimer's disease (AD), an age‐associated disease. The expression patterns of IQCK in healthy and AD brains, within the context of age and sex are largely unknown. Therefore, we compared the age‐dependent expression patterns of IQCK in males and females of wild‐type (WT) mice with AD‐like 3xTg and APΔE9 mice. Additionally, we measured IQCK protein expression in AD‐derived human iPSC neurons. In WT mice, we found no IQCK expression at day 1 (1D) in the cortex (CX), hippocampus (HP), brainstem (BS) and cerebellum (CB). Overall, IQCK protein expression in different brain regions was first detected in 1‐month‐old wild‐type (WT) mice, reaching its maximum in 1‐year‐old mice (1Y), and then gradually decreased in 2‐year‐old mice. In the APΔE9 mice, IQCK protein levels significantly increased by 1246% in the CX, 682% in the HP and 169% in the BS relative to WT controls. In the 3xTg mice, only HP showed an increase of IQCK protein by 277%. In addition, we also detected elevated tendencies in BS and CB regions but not in the CX. Finally, IQCK expression was also significantly increased by 68% in the AD‐derived iPSC neurons relative to the NC‐derived iPSC neurons. Thus, increased IQCK protein levels in the brain of AD‐like 3xTg and APΔE9 mouse models suggest a possible role in AD pathogenesis, a finding that requires further clarification.

## 1 Introduction

Aging is the single greatest cause of disease and death worldwide, and understanding the molecular basis for aging could vastly improve quality of life. The brain, in particular, is vulnerable during normal aging, resulting in various behavioral changes, including an overall decline in cognitive function, especially a reduction of short‐term [1] and spatial memory [2]. However, the underlying molecular mechanisms for decreased brain function are still unclear. Any protein's quantity at any given time depends on its turnover, ranging from minutes to several days. It is regulated by multiple factors and mechanisms, including ubiquitin‐proteasome and autophagy‐mediated degradation [3, 4, 5]. Notably, protein perturbation turnover is known to cause severe neurological dysfunction [6]. Indeed, Alzheimer's disease (AD) is characterized by imbalances in the turnover of a few proteins, resulting in their accumulation into misfolded protein aggregates [7, 8, 9]. Therefore, quantifying key proteins during aging and AD and their differential expression is crucial in unraveling disease‐contributing proteins. Age is the greatest risk factor for AD [10, 11]. It is expected that over 100 million people worldwide will be living with dementia by 2050 [12], with an estimated cost to reach $1 trillion in a few years [13]. AD is the leading cause of dementia. The risk of developing AD roughly doubles every 5 years after 65, and it is close to 50% over the age of 85, clearly suggesting that age is the greatest risk factor for AD [14, 15].

Although plaques and tangles are the hallmark features of AD, synapses are now considered early sites of dysfunction [16, 17, 18], and loss of synapses is the best pathological correlate of memory loss in AD patients [19, 20]. Interestingly, marked alterations of synapses have been detected in the brain of patients, even in the very early stage of AD or with mild cognitive impairment (MCI), when there is little or mild amyloid‐beta (Aβ) plaques and neurofibrillary tangles or neuronal death [21]. Loss of synapses and the disintegration of neural networks is the basis for decreased signal transmission strength and efficiency, leading to cognitive decline. Accordingly, AD is considered a synaptic plasticity failure [22]. So, it is also crucial to identify and target precise molecules and pathways responsible for synaptic damage in AD.

In recent years many genome‐wide association studies (GWAS) were actively pursued to disentangle the genetic underpinnings of late‐onset Alzheimer's disease (LOAD). The GWAS studies, which used larger sample sizes of more than a million cases across multiple populations and proxy phenotypes from biobanks, have so far increased the number of known susceptibility loci in AD to more than 80 and 90 independent variants across 75 ad and dementia susceptibility loci [23, 24, 25, 26]. These multiple studies have reinforced the importance of amyloid precursor protein (APP) and tau metabolism in the cause of the disease and immune response, inflammation, lipid metabolism, endocytosis as well as intracellular trafficking and cell migration in the cause of the disease. While these studies have advanced our understanding of the genetic architecture of AD, the next great challenge lies in prioritizing those genes that modulate disease susceptibility and nominating candidate causal genes for further validation in both cellular and animal models.

Using stringent quality control protocols, recent GWAS studies found both a common missense variant and a relatively common splicing variant of novel IQ‐motif‐containing protein K (IQCK) to increase the risk of AD [27, 28, 29, 30]. In addition, it was also demonstrated that IQCK increases AD risk regardless of APOE ε4 status [31], suggesting that IQCK may contribute to AD in a lipid metabolism‐independent manner. It is also essential to emphasize that IQCK serves as a risk factor for obesity [27, 30] and obsessive‐compulsive disorder (OCD) [32], which are also known to increase the risk of AD [33, 34, 35]. Parallel studies also confirmed IQCK mutation in congenital anomaly limb body wall complex (LBWC) [36] and a robustly increased circular IQCK RNA but not linear RNA in the multiple system atrophy (MSA) brains, especially in the white matter of the frontal cortex [37]. This evidence strongly supports the crucial role of IQCK in neurons, the brain, and likely in AD.

Although the exact function of IQCK is currently unknown, IQ‐motif is a known site for binding to different calcium‐binding proteins such as calmodulin, and knockdown of IQCK mRNA in the zebrafish has shown ventral defects, including failure to develop the ventral fin as well as cardiac edema [36]. In our previous study, we demonstrated IQCK expression in neurons including neuritic processes, astrocytes and oligodendrocytes, but not in microglia [38]. Therefore, we aimed to investigate changes in IQCK expression during brain aging and AD pathology using two experimental AD models and corresponding wild‐type controls. Finally, we addressed whether IQCK expression in iPSC‐derived neurons colocalizes with the spine marker spinophilin.

## 2 Materials and Methods

### 2.1 Chemicals and Antibodies

The sodium orthovanadate (cat # 450243), dithiothreitol (cat # D9779) and protease inhibitor cocktail (cat # P8340) were purchased from Sigma Aldrich (St. Louis, MO, USA). Nonidet‐P40 substitute (cat # M158) for lysis buffer preparation was obtained from Amresco (Solon, OH, USA). Microcystin‐LR (cat# 475815) was purchased from Calbiochem‐Millipore (Temecula, CA, USA). Syn‐PER Synaptic Protein Extraction Reagent (cat # 87793) was purchased from Thermo Fisher Scientific (Waltham, MA, USA). USDA‐certified fetal bovine serum (FBS) for cell cultures was purchased from BioFluid Technologies (cat # SKU: 100‐500‐Q). SuperSignal West Pico PLUS Chemiluminescent Substrate (cat # 34578) and PageRuler Prestained Protein Ladder, 10–180 kDa (cat # 26617) were purchased from Thermo Fisher Scientific. Polyclonal IQCK antibody was purchased from Abbexa (cat # abx234377). The rabbit polyclonal antibody CT15, which reacts with the C‐terminal 15‐aa residues of APP, was used for the detection of full‐length APP and has been described previously [39, 40, 41]. Polyclonal spinophilin antibody was purchased from Cell Signalling (cat # 9061). Monoclonal beta actin antibody (C4) (cat # sc‐47778) was purchased from Santa Cruz Biotechnology Inc. (Dallas, TX, USA). Secondary antibodies such as peroxidase‐conjugated AffiniPure goat anti‐rabbit (code # 111‐035‐144) IgG (H + L) and goat anti‐mouse (Code # 115‐035‐146) were purchased from Jackson ImmunoResearch Laboratories (West Grove, PA, USA). The Donkey F(ab')2 anti‐mouse IgG H&L (Alexa Fluor 568) for immunocytochemical staining was purchased from Abcam (cat # ab175699). DAPI Fluormount‐G (cat # 0100‐20) for mounting slides was purchased from Southern Biotech (Birmingham, AL, USA). For immunoblot analysis, a 5% Americanbio Inc. non‐fat dry milk (cat # NC0115668), Fisher Scientific (Waltham, MA, USA) prepared in tris‐buffered saline with 0.1% Tween‐20 (TBS‐T) was used to dilute all the primary antibodies, while the secondary antibodies were diluted directly in the TBS‐T buffer.

### 2.2 Quantification of Proteins by Western Blotting

We strictly followed the National Institute of Health's ‘Guide for the Care and Use of Animals’ and used approved protocols by the Florida International University's Animal Care and Use Committee (IACUC‐21‐022‐CR02). We obtained AD‐like 3xTg (cat # 034830) and APΔE9 mice (cat # 034829) from Jackson Laboratories. The 3xTg mice are homozygous for all three mutant alleles homozygous for the Psen1 mutation and homozygous for the co‐injected APPSwe and tauP301L transgenes (Tg(APPSwe,tauP301L)1Lfa, MMRRC stock #34830) [42]. APΔE9 mice (MMRRC Strain #034833‐JAX) overexpress chimeric mouse/human APP (Mo/HuAPP695swe) and a mutant human presenilin 1 (PS1‐ΔE9), both transgenes driven by independent prion promoters [43]. The genotype of the APΔE9 mice was confirmed initially by genotyping the tail genomic DNA and PCR analysis using specific primers. To assess age‐associated changes of IQCK protein expression in the mouse brain, we used both male and female C57BL/6 mice (Wild‐type, WT) of 1‐day (1D), 1 month (1M), 1 year (1Y), 1.5 years (1.5Y) and 2 years old (2Y) mice(*n* = 3/genotype/age group). We further compared the IQCK protein levels in the brain of AD‐like 1 year‐old 3xTg and 1.5‐year‐old APΔE9 mice with age‐matched WT mice.

The mice were euthanized by carbon dioxide overdose, decapitated immediately and different brain regions such as cortex (CX), hippocampus (HP), brainstem (BS) and cerebellum (CB) were rapidly dissected and separated on ice and placed into Syn‐PER Synaptic Protein Extraction solution with complete protease inhibitor mix supplemented with sodium vanadate and microcystin. Following uniform homogenization of all samples, they were centrifugated at 14,000 rpm for 20 min at 4°C. The lysate samples containing about 30 micrograms of protein were mixed with equal amounts of loading buffer, loaded into each well, and subjected to SDS‐PAGE electrophoresis exactly as described previously [38, 44, 45]. The proteins were then transferred onto PVDF membranes, blocked with 5% milk prepared in 1% TBS‐T buffer, and incubated overnight with primary antibodies followed by 1‐ to 2‐h incubation with HRP‐conjugated anti‐mouse or anti‐rabbit secondary antibodies. The protein signals were detected at different exposure times following incubation with the SuperSignal West Pico chemiluminescent substrate (cat # 34580, ThermoFisher, USA). Quantification of Western blot signals was done using freely available NIH ImageJ software. Actin signals were used to normalize protein levels in each sample and the protein levels were shown in percent of wild‐type (WT) controls.

### 2.3 iPSC‐Derived Neuronal Cultures

Human iPSC‐neural stem cells (NSCs) derived from dermal fibroblasts of a single AD patient with presenilin mutation (PSEN1 A246E) (cat # ax0114) and normal control (NC) human iPSC‐neural stem cells (cat # ax0018) derived from a single subject were purchased from Axol Bioscience Inc. We cultured cells following the manufacturer's recommended protocol. Briefly, cell culture dishes were coated with SureBond (cat # ax0053) at 37°C for 4 h to promote attachment and growth of neural stem cells. The cryopreserved NSCs were thawed rapidly, mixed with neuronal medium, plated and cultured in a humidified incubator with 5% CO_2_ at 37°C. Next, the neuronal maintenance medium was supplemented with 1% Glutamax, 2% B27 Supplement, epidermal growth factor at 20 ng/mL concentration, and basic fibroblast growth factor at 20 ng/mL concentration. The medium was changed three times per week and maintained up to 4 weeks in culture.

### 2.4 Immunocytochemical Staining of iPSC Neurons

We used neurons at 16 days‐in vitro (16DIV) on specially coated coverslips and then subjected them to immunocytochemical staining. Neurons were washed in cold phosphate‐buffered saline (PBS), fixed in 4% paraformaldehyde (PFA) in PBS for 10 min, and washed three times in tris‐buffered saline with 0.1% Tween 20 detergent (TBST). Cell permeabilization was carried out using 0.4% Triton X‐100 for 5 min and then blocked with a blocking solution (normal goat serum, 1%; BSA, 3%; gelatin, 1%; Triton X‐100, 0.2%; saponin, 0.2%) for 30 min and then incubated with spinophilin primary antibody overnight, followed by incubation with Alexa Fluor 568‐conjugated anti‐rabbit IgG secondary antibody for 1 h followed by mounting with 4′,6‐diamidino‐2‐phenylindole (DAPI) containing Fluormount‐G (SouthernBiotech, Birmingham, AL) to visualize the nuclei. Neurons positively stained with various primary antibodies such as IQCK, NeuN, doublecortin (DCX), spinophilin, MAP2, AT100 for phosphorylated tau and CT15/63D for amyloid precursor protein (APP) were identified on the green channel (GFP) or at 568 red channel (Alexa Fluor 568) and were captured in a BZX700 All‐in‐One microscopy system (Keyence Corp, Itaska, IL, USA). The IQCK‐positive neurons from AD were randomly selected to quantify IQCK fluorescence intensity and compared with those from NC‐derived neurons. We used an equal number of 100 neurons for each NC and AD for quantification. ImageJ was used to quantify the IQCK fluorescence intensity, first by converting the images to RGB color mode, the scale was set in pixels, the area of the neurons for measurement was selected using a freehand line, and the fluorescence intensity was measured. We used random selection criteria, and the selected neurons should be completely independent and should have a DAPI‐positive nucleus.

### 2.5 Statistical Analysis

ImageJ software from NIH was used to analyze and quantify immunolabelled proteins detected by Western blot analysis. All protein levels were normalized to β‐actin levels to reflect true changes in each sample. All statistical analyses were performed using the Instat3 software (GraphPad, San Diego, CA, USA). For comparisons in the levels of IQCK between two groups such as WT versus 3xTg or WT versus APΔE9, the student's two‐tail paired t‐test was used. A one‐way analysis of variance (ANOVA) followed by Dunnett's test was used to compare IQCK protein levels among the different age groups in multiple brain regions. Student's t‐test was used to quantify IQCK immunofluorescence intensity in the iPSC neurons derived from NC and AD. Data presented are the mean ± standard error of the mean (SEM) and were considered significant only if *p* < 0.05. ** indicates *p* < 0.01, *** indicates *p* < 0.001 and **** indicates *p* < 0.0001.

## 3 Results

### 3.1 Age‐Dependent Differential Expression of IQCK in Different Regions of the Mouse Brain

**FIGURE 1:**
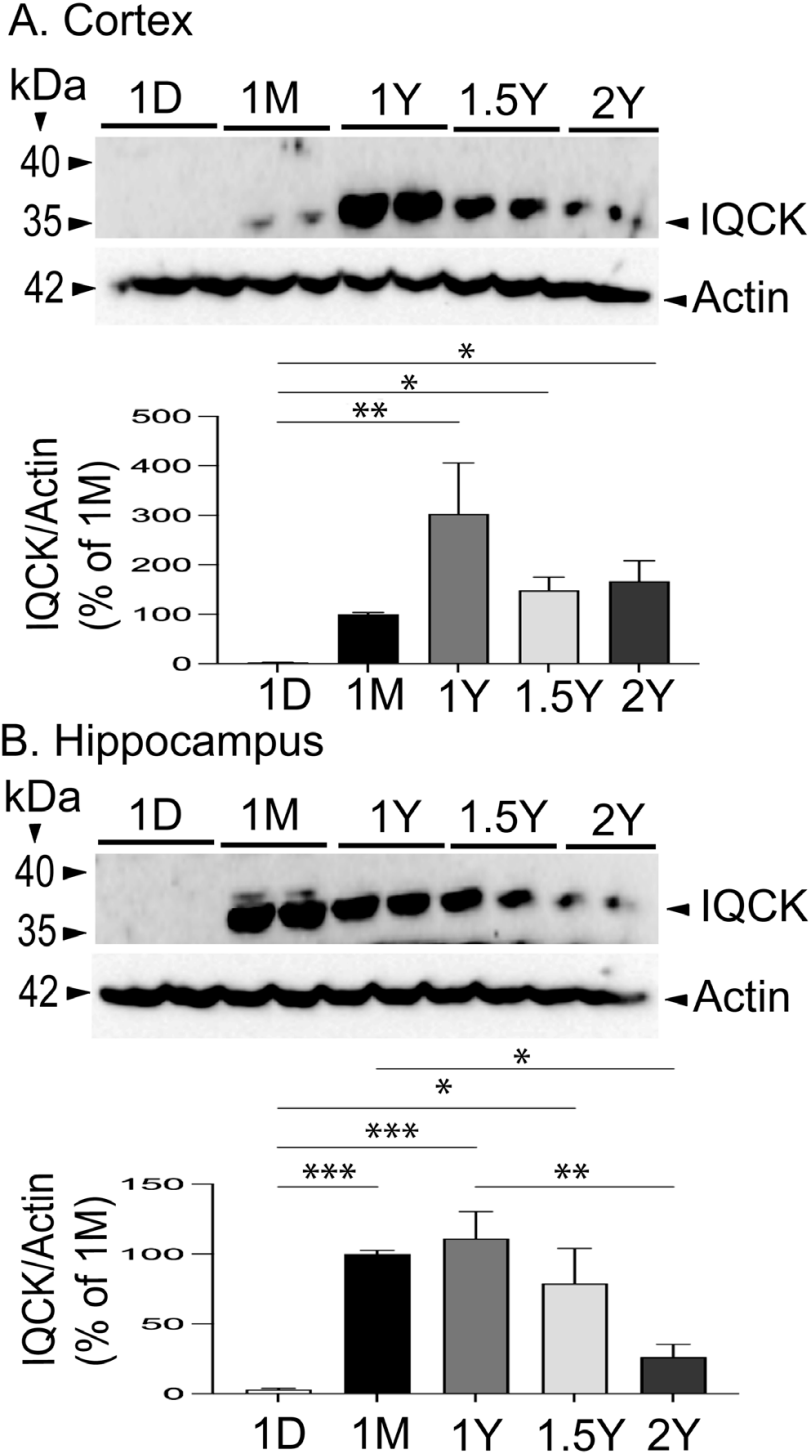
Age‐dependent variations in the expression of IQCK protein in the cortex (CX) and hippocampus (HP) of wild‐type (WT) mice at 1 day (1D), 1 month (1M), 1 year (1Y), 1.5 years (1.5Y) and 2 years (2Y). In the CX, IQCK protein expression is absent at 1D, increases to maximum levels at 1Y, and then gradually reduces by 2Y. In the HP, IQCK protein levels reached a maximum at 1 year and then gradually decreased at other ages. Data were statistically analyzed by Analysis of Variance (ANOVA) followed by the Tukey–Kramer Multiple Comparisons Test. *, *p* < 0.05, **, *p* < 0.01 and ***, *p* < 0.001, Data are mean ± SEM, *n* = 3 per group.

**FIGURE 2:**
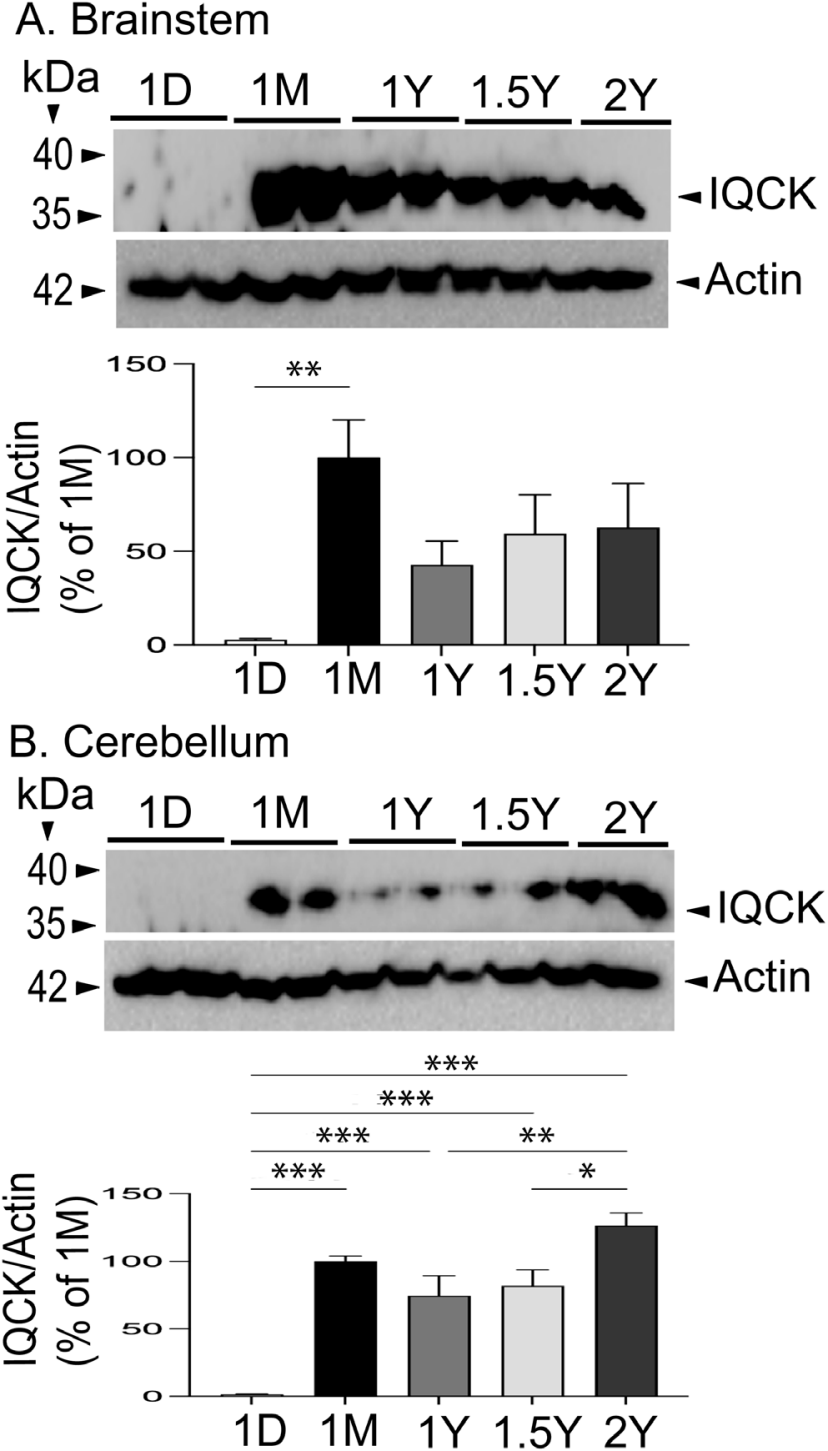
Age‐dependent variations in the expression of IQCK protein in the brainstem (BS) and cerebellum (CB) of wild‐type (WT) mice at 1 day (1D), 1 month (1M), 1 year (1Y), 1.5 years (1.5Y) and 2 years (2Y). In the BS, IQCK protein expression is absent at 1D, increases to maximum levels at 1M, and then is expressed at lower levels at other ages. In the CB, no IQCK protein expression was observed at 1D, followed by comparable expression at 1M, 1Y and 1.5Y, with the highest expression at 2Y. Data were statistically analyzed by Analysis of Variance (ANOVA) followed by the Tukey–Kramer Multiple Comparisons Test. *, *p* < 0.05, **, *p* < 0.01 and ***, *p* < 0.001. Data are mean ± SEM, *n* = 3 per group.

The quantity and differential expression of a given protein at different ages are likely to shed light on its function. Also, since multiple brain regions exhibit diverse heterogeneity in both structure and function, it is important to understand the brain region heterogeneity in protein expression. As there is no information on IQCK expression during aging or in different brain regions, we quantified IQCK protein levels in the cortex (CX), hippocampus (HP), brainstem (BS) and cerebellum (CB) at different ages such as 1 day (1D), 1 month (1M), 1 year (1Y), 1.5 years (1.5Y) and 2 years (2Y) in the C57BL/6 wild‐type (WT) mice. We found that IQCK protein is not expressed in 1D‐old pups in any of the brain regions studied such as CX. HP, BS and CB (Figures 1 and 2).

Since 1M was the earliest IQCK expression first detected, we used 1M IQCK protein levels to compare relative expression across the ages. Thus, in the CX, IQCK expression levels were 303% (*p* < 0.01), 148% (*p* < 0.05) and 167% (*p* < 0.05) at 1Y, 1.5Y and 2Y, respectively (Figure 1A). Similarly, relative to IQCK protein levels at 1M in the HP, the levels were significantly increased by 111%, 79% and 26% in the 1Y, 1.5Y and 2Y ages, respectively (Figure 1B). In the BS, the IQCK expression levels were 43%, 60% and 63% at 1Y, 1.5Y and 2Y, respectively, relative to their expression levels at 1M (Figure 2A). Finally, in the CB, the expression levels were 74%, 82% and 126% of those at 1M in the 1Y, 1.5Y and 2Y age groups, respectively (Figure 2B). In the CX, there was no expression at 1D, but IQCK expression increases at 1M and reaches a maximum at 1Y, and then gradually reduced at 1.5Y and 2Y time points. In the HP, there is an elevated tendency to increase from 1M to 1Y, and then it is reduced at 1.5Y and 2Y. In the BS, the maximum is reached at 1M and then reduced throughout the other ages. In the CB, the increase is observed at 1M and remains relatively constant at 1Y and 1.5Y, before increasing again at 2Y.

Based on the results presented in Figures 1 and 2, at 1D none of the brain regions examined showed any expression of IQCK. At 1M, IQCK expression is low in the CX, relatively high in the HP, and highest in the BS followed by a relatively good expression in the CB. At 1Y, IQCK expression is highest in the CX and HP, followed by relatively low expression in the BS and CB. At 1.5Y, IQCK expression is relatively higher in both the BS and CB compared to relatively lower IQCK expression in the CX and HP. Finally, at 2Y, both CX and HP showed weak IQCK expression, whereas BS showed moderate expression levels and CB showed relatively high IQCK levels. Thus, overall, in all brain regions studied there is no IQCK expression at 1D and then at 2Y also the protein expression is reduced particularly in CX and HP compared to 1M, 1.5Y and 2Y time points.

### 3.2 IQCK Protein Levels Are Robustly Increased in the APΔE9 Mouse Model of AD

**FIGURE 3:**
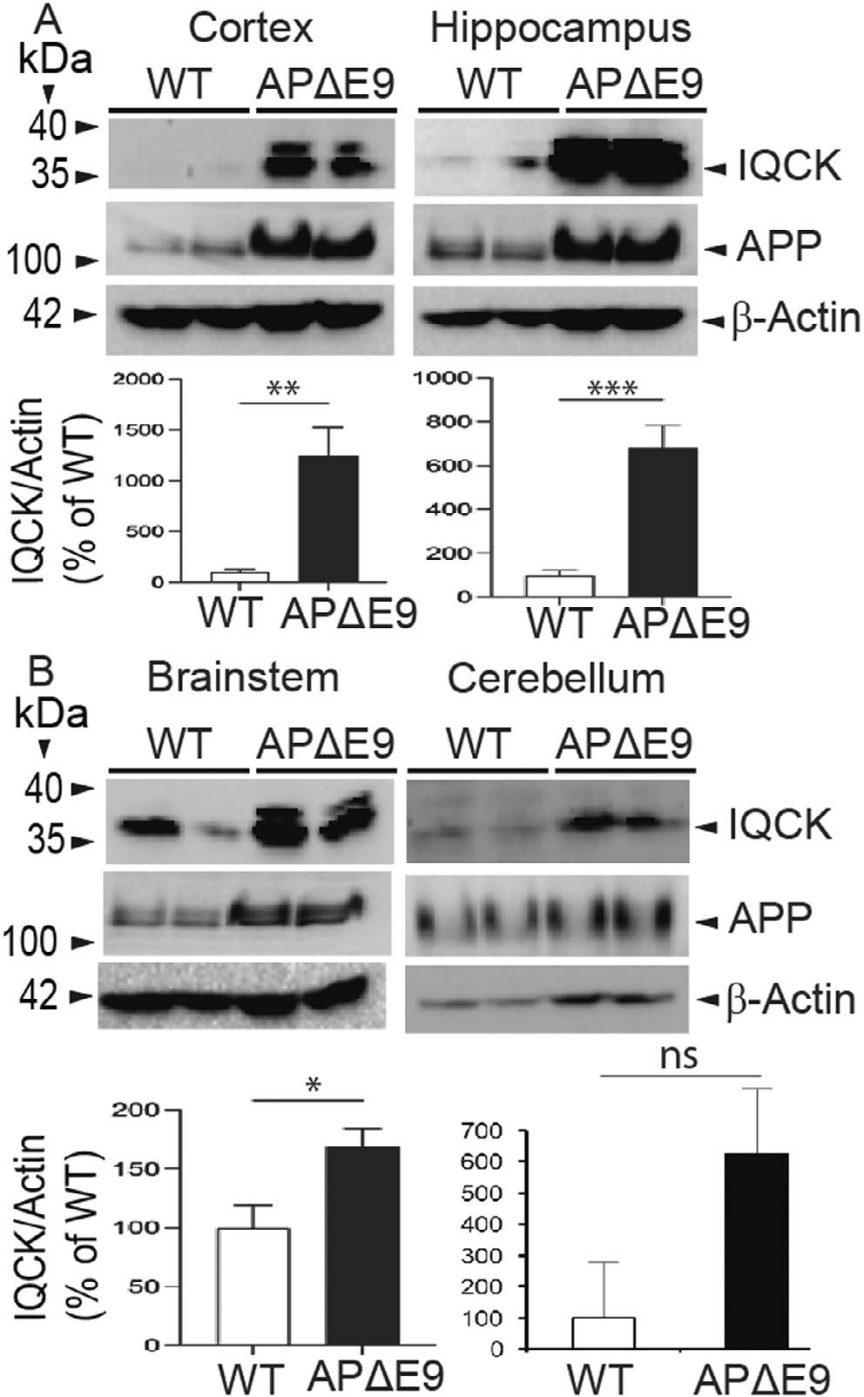
IQCK protein is significantly increased in different regions of the brain in the APΔE9 mouse model of AD relative to wild‐type (WT; non‐transgenic) controls, as detected by western blot assay. IQCK protein levels were normalized to Actin levels, used as loading controls, and quantified using ImageJ, and then compared among APΔE9 and WT control mice. APP expression is shown to validate the APΔE9 mice relative to WT controls. The APΔE9 cortex (CX) showed 1246% increased IQCK levels compared to WT mice. The hippocampus (HP) showed a 682% increase while the brainstem (BS) showed a 169% increase in the APΔE9 mice relative to WT controls. Data were statistically analyzed by paired t‐test. *, *p* < 0.05, **, *p* < 0.01 and ***, *p* < 0.001. Data are mean ± SEM, *n* = 3 per group.

Since we recently found that IQCK protein levels were significantly increased in the AD patient brains relative to normal control brains [38], we next aimed to verify whether IQCK protein levels are altered in the mouse brain using an experimental APΔE9 model of AD. These transgenic mice overexpress chimeric mouse/human APP (Mo/HuAPP695swe) and a mutant human presenilin 1 (PS1‐ΔE9), both transgenes driven by independent prion promoters [43] Because this transgenic line starts depositing plaques as early as six months and starts secreting Aβ within 3–4 months [46], this mouse line is a good model of early‐onset AD. Quantification revealed that IQCK protein levels were significantly and robustly increased in the CX by 1246% (**, *p* < 0.01), HP by 682% (***, *p* < 0.001) and in BS by 169% (*, *p* < 0.05) in the APΔE9 brains when compared to the WT brains (Figure 3). However, in the CB, although the increase was 6‐fold it was not statistically significant due to high SEM (Figure 3). To validate these results, we also probed the blots with CT15 antibody which detects APP [39, 40, 41]. As shown in Figure 3 expression of APP is high when compared to WT in all the brain regions, confirming that the increased IQCK protein levels in different brain regions occur in the APΔE9 brains relative to WT brains.

### 3.3 IQCK Is Increased in the Hippocampus of the 3xTg Model of AD

**FIGURE 4:**
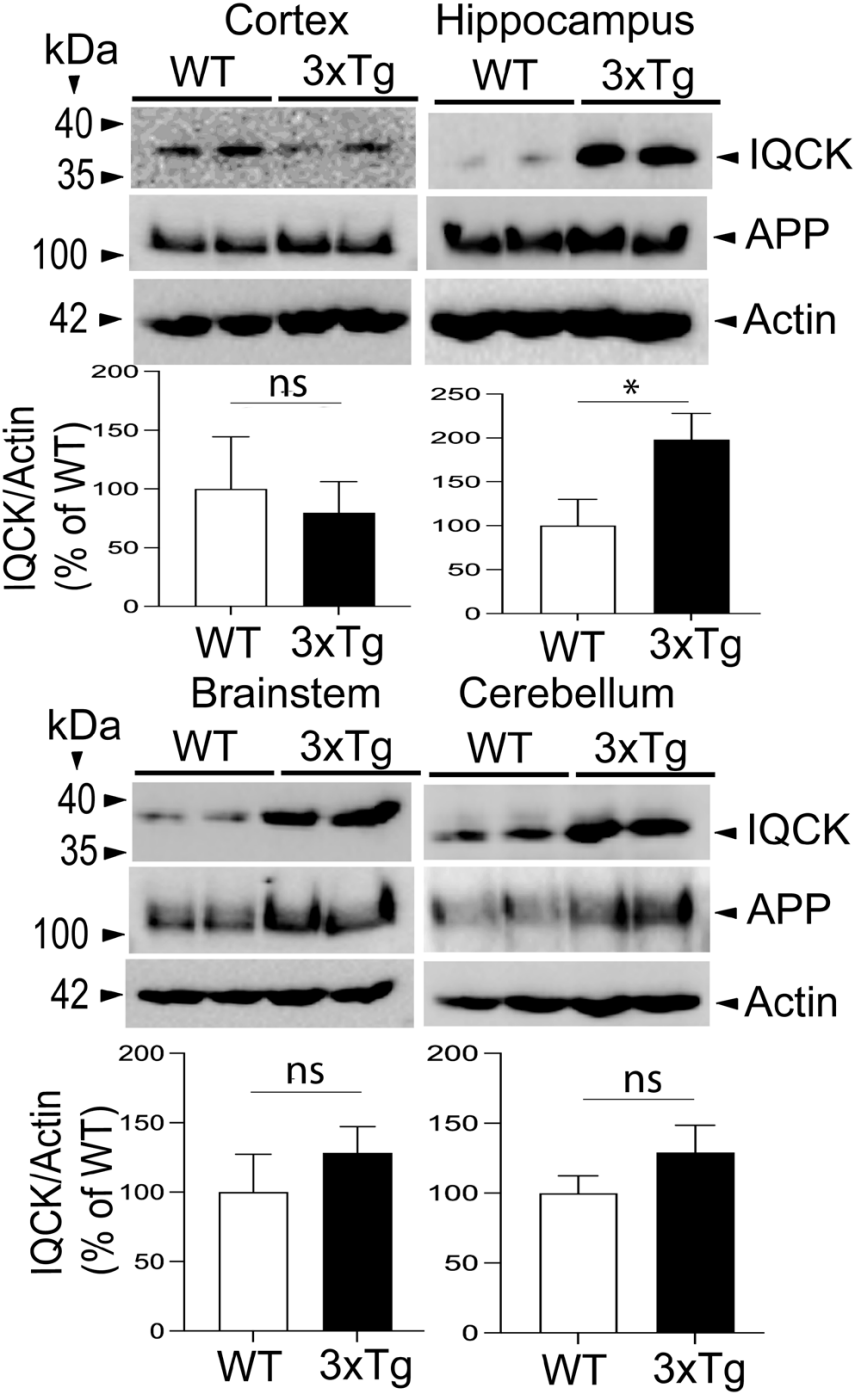
IQCK protein is significantly increased in the hippocampus brain region of 3xTg AD‐like mice relative to the wild‐type (WT; non‐transgenic) controls, as detected by western blot assay. IQCK protein levels were normalized to Actin levels used as loading controls, quantified by ImageJ, and compared among 3xTg and WT control mice. APP expression is also shown to validate the 3xTg mice relative to WT controls. Relative to WT, the 3xTg hippocampus (HP) showed a 277% increase in IQCK levels. The other brain regions showed an increased trend but were not significant. Data were statistically analyzed by paired t‐test. *, *p* < 0.05. Data are mean ± SEM, *n* = 3 per group.

Since we confirmed elevated IQCK protein expression in the APΔE9 model of AD, we next tested whether the increase in IQCK is specific to one model of AD or shared to other models. Therefore, we also measured IQCK protein levels in the 3xTg mice. Unlike APΔE9 mice, where only APP and presenilin mutations are driven to express, 3xTg mice also express tau P301L transgene. Thus, these mice are valuable for studying the impact of both amyloid and tau pathology [42, 43]. Results revealed that, similar to APΔE9 mice, HP showed a significant increase of IQCK protein by 277% (*, *p* < 0.05) in the 3xTg mice compared to WT controls (Figure 4). Furthermore, no or minor significant alteration in IQCK levels was observed in the brain CX region of 3xTg mice relative to age‐matched WT mice. However, the BS and CB brain regions showed an elevated, non‐significant tendency of IQCK expression. Altogether, these data indicate increased IQCK protein in the HP brain region of both models. Furthermore, the APΔE9 model also demonstrates increased IQCK protein in the CX and BS. The CB is the only brain region among the areas studied where the IQCK protein was not significantly altered. Our results also suggest that IQCK expression is upregulated independently of Tau phosphorylation, as only 3xTg mice exhibit additional tau‐associated AD pathology compared to APΔE9 mice. In contrast, it is plausible that an increased IQCK protein level is due to the APP mutation, as both 3xTg and APΔE9 mice express human mutant APP linked with AD.

### 3.4 Healthy Control and AD Patient‐Derived iPSC Neurons

**FIGURE 5:**
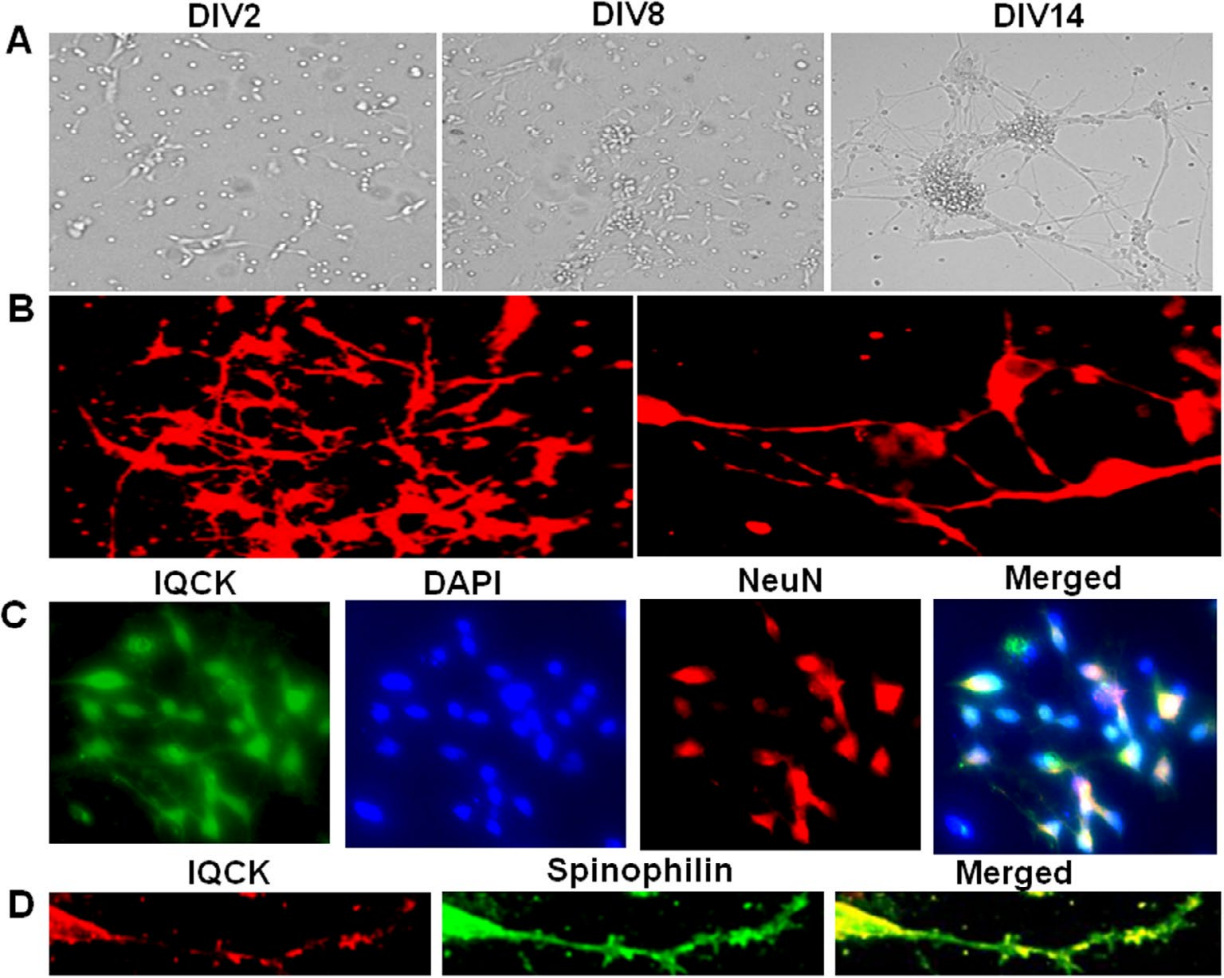
IQCK protein is expressed in progressively networking iPSC‐derived NeuN‐confirmed neurons and MAP2 and spinophilin confirmed dendritic spines. iPSC cells were grown and differentiated into neurons for 16 days in vitro (16DIV) and then immunocytochemically stained for IQCK, along with NeuN or Spinophilin, as markers for neurons or dendritic spines, respectively. (A) The gradual formation of a complex network of neurons is shown on DIV2, DIV8 and DIV14. (B) Staining with MAP2 antibody shows a complex network of dendrites. (C) IQCK protein is expressed in mature NeuN^+^ neurons. (D) IQCK protein is expressed in the dendritic spines, as supported by the merged images of IQCK and spinophilin.

We monitored iPSC neurons every day for the formation of a complex network of neuritis and found that in the initial days of plating, there was no neuritis, and gradually by 8DIV, long lengths of neuritis could be seen in most of the neurons, and by 14DIV, a complex network of neuritis could be observed (Figure 5A). Initially, the neural cells were positive for the doublecortin (DCX), a microtubule‐associated protein considered the marker of neural progenitor cells. By 16DIV, neurons were immunoreactive to MAP2 and showed a complex network of mature dendrites (Figure 5B). Importantly, by 16DIV neurons were also positively stained for NeuN, a marker of mature neurons. Interestingly, coimmunostaining also revealed that IQCK protein is expressed in neurons (Figure 5C). We previously found that IQCK is robustly expressed within the neuronal processes in the brain as well as in primary neurons [38]. Remarkably, these high magnification images also revealed that IQCK protein is expressed in the dendritic spines as judged from the merged images in Figure 5D; co‐stained with antibodies against IQCK and spinophilin, a well‐established marker of dendritic spines. Thus, we confirmed that IQCK is expressed in iPSC‐derived mature neurons and that IQCK is also expressed in the dendritic spines. Notably, our aim was to determine any expression change of IQCK in the neural networks formed by iPSC neurons from the AD patients and those from healthy controls at different time points in vitro. However, it remains unclear whether differences exist in dendritic organization and the number of dendritic spines. Future studies will address these questions.

### 3.5 IQCK Protein Expression Is Significantly Increased in AD‐Derived iPSC Neurons

**FIGURE 6:**
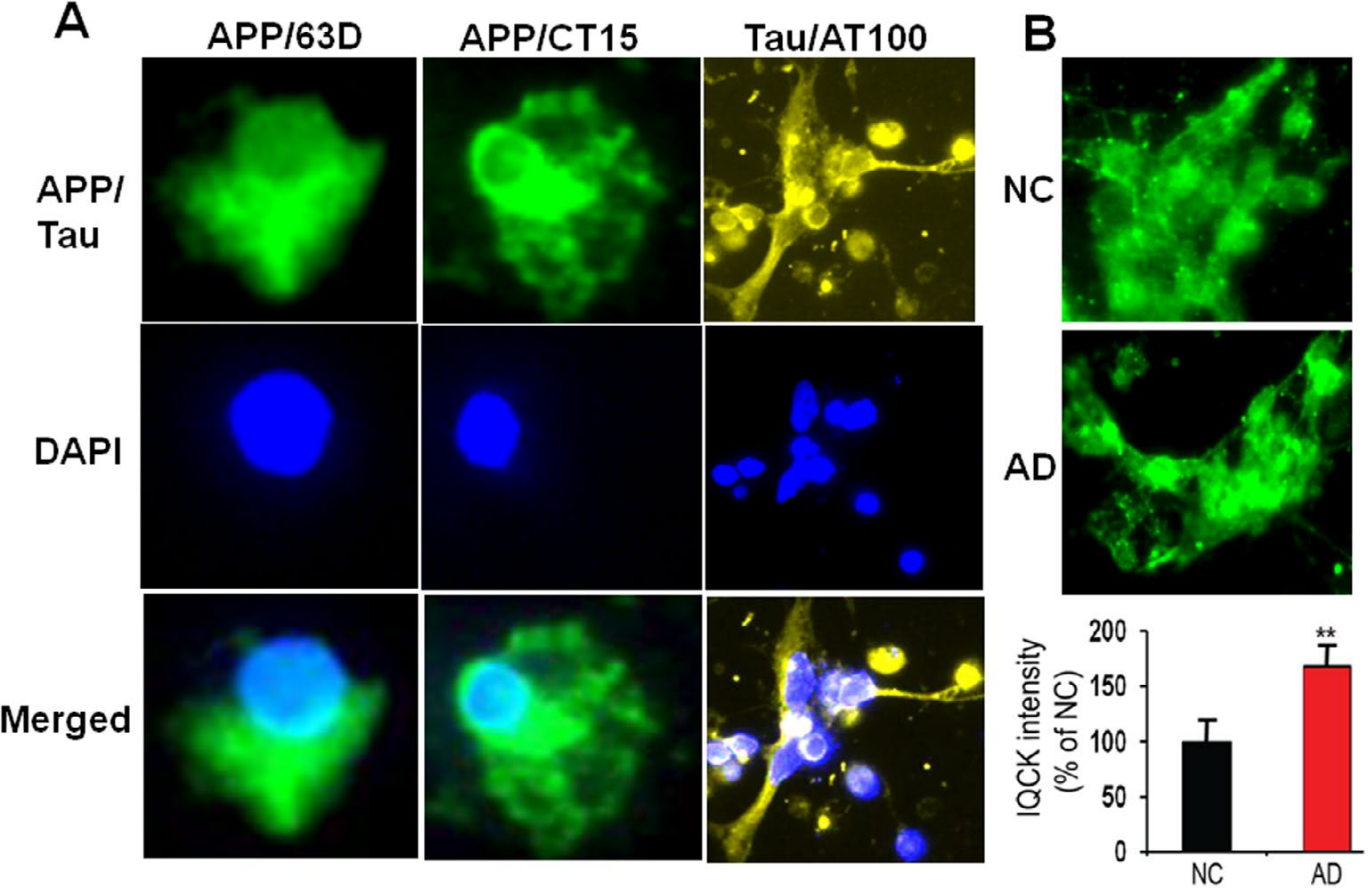
IQCK protein expression is increased in iPSC‐derived neurons from AD patients relative to NC‐derived neurons. (A) iPSC neurons express APP and phosphor‐tau proteins. Confirmation of APP expression with two antibodies, 63D and CT15, and expression of phospho‐tau with AT100 antibody in iPSC‐derived neurons. (B) IQCK expression is increased in AD‐derived iPSC neurons relative to iPSC neurons derived from healthy controls. Quantification of IQCK immunofluorescence showed increased levels in AD‐derived iPSC neurons when compared to NC‐derived neurons. Data were statistically analyzed by paired t‐test. **, *p* < 0.01, Data are mean ± SEM, *n* = 4 per group.

To be relevant to AD as a neuronal cellular model, we next aimed to confirm whether iPSC neurons express AD‐related proteins such as APP and tau. In Figure 6A we show that a typical membranous APP immunoreactivity could be seen when neurons were stained with CT15 antibody (raised against the last 15 residues of APP). To confirm this, we further used a different anti‐APP 63D antibody (raised against APP ectodomain). Our data detected a similar immunoreactivity of APP in the plasma membranes of mature neurons stained with 63D antibody. Similarly, staining of neurons with AT100 antibody revealed a significant staining of neurons, suggesting that these mature iPSC neurons express phosphorylated tau (Figure 6A). Thus, our results support that iPSC‐derived mature neurons express both APP and tau relevant to AD. Even more importantly, quantification of IQCK immunofluorescence intensity showed a significantly increased level (68%, *p* < 0.01) in the AD‐derived iPSC neurons when compared to NC‐derived iPSC neurons (Figure 6B). These results are consistent with those shown in two AD mouse models (Figures 3 and 4) and as we previously demonstrated in the human brain [38]. However, the relevance of the increased IQCK protein in AD models relative to NC needs to be further investigated.

## 4 Discussion

The proportion of risk attributable to genetic susceptibility factors for AD has been estimated to be between 60% and 80% and so far, genome‐wide association studies (GWASs) have identified more than 80 ad‐associated genes. The greatest challenge for future therapeutics is characterizing how these novel risk genes cause AD. In the current study, we found that IQCK expression is both age‐ and brain region‐dependent, and importantly IQCK protein expression is robustly increased in two mouse models of AD and also in iPSC neurons derived from dermal fibroblasts of an AD patient.

Aging is associated with a gradual decline in the overall function of an organism, which is tightly linked to changes in the expression of specific proteins. Indeed, dysregulation of proteins associated with neuroinflammation and neurodegeneration contributes to age‐associated AD [47, 48]. Since IQCK is a novel risk factor for AD and other diseases [27, 28, 29, 30, 31], and its age‐associated expression is unknown, we investigated IQCK expression as a function of age. Remarkably, our results clearly suggest that IQCK is not required during postnatal brain growth, is robustly expressed during adulthood, and then surprisingly decreases in old age, especially in brain regions such as CX and HP but not CB where IQCK expression increased at the 2‐year time point. Interestingly, although IQCK expression decreased during physiological aging in the CX and HP, both APΔE9 and 3xTg mouse models of AD showed a robust elevation in IQCK expression in the HP brain region. The APΔE9 mice also showed increased IQCK in the CX and BS. In recent years, several investigators have noted that 3xTg mice are losing their phenotype. We also noted that the APP expression levels in most brain regions of 3xTg mice were almost comparable to WT control brains. This may account for unchanged IQCK levels in the CX and BS regions in the 3xTg mouse brains. In the APΔE9 mice, IQCK levels were robustly increased in the same brain regions. Furthermore, when IQCK expression levels are compared among different brain regions at a specific time point, we note that: (I) there is no IQCK expression at 1D; (II) IQCK is expressed highest in the BS, lowest in the CX and at comparable levels in the HP and CB by 1M. In the adult at 1Y age, IQCK is expressed at its highest in the CX and HP, but both BS and CB also express relatively high levels of IQCK protein. Interestingly, both CX and HP express relatively lower levels compared to BS and CB in the older 1.5‐year age group. At the 2Y time point, CB further expresses the highest IQCK protein levels compared to all other brain regions. Therefore, during adult ages, IQCK expression is particularly high in the CX and HP brain regions. The present results cannot be compared since IQCK is mostly an uncharacterized novel protein. However, we recently demonstrated that IQCK protein levels were markedly increased in the AD patient brains compared to normal controls by immunoblots, which were also supported by immunohistochemical staining [38]. Importantly, increased IQCK immunoreactivity was found within the amyloid plaques, implying that IQCK may have an important causative role in AD. Therefore, our preclinical data may suggest a pertinent role of IQCK in AD pathology.

Another interesting finding of the present study is a significant increase of the IQCK protein expression in the AD patient‐derived iPSC neurons. It is now well established that neurons derived from iPSCs carrying FAD mutations show AD‐associated pathologies, including Aβ secretion [49, 50, 51]. Thus, these neurons do not require exogenous expression of mutant proteins to study the role of specific proteins on Alzheimer's pathology. The iPSCs that we used were derived from dermal fibroblasts of an AD patient with presenilin (PSEN1 A246E) mutation. Since these neurons are mature, as reflected by NeuN positivity with a complex network of dendrites as reflected by MAP‐2, the increased IQCK immunoreactivity can be interpreted to result from presenilin mutation‐induced AD pathology.

## 5 Conclusions and Future Directions

Because IQCK levels are reduced during physiological aging in WT mouse brains (Figures 1 and 2), the increased IQCK levels in experimental models of AD and AD patient‐derived iPSC neurons (Figures 3, 4, 5, 6) may positively correlate with the loss of dendritic spines. Interestingly, IQ‐motif‐containing proteins are known to bind to calmodulin [52], a key regulator of intracellular calcium in the dendritic spines, which is crucial for the induction of long‐term potentiation (LTP) and long‐term depression (LTD) for synaptic plasticity [53, 54]. The other IQ proteins, such as IQSEC2 [55], IQSEK3 [56] and IQGAP1 [57], have also been shown to play an essential role in dendritic spine regulation. We also previously demonstrated a pivotal role of IQCK expression in neuronal processes using primary neurons and in the mouse brain [38]. Thus, the finding that IQCK is also expressed in the dendritic spines aligns with other members of its family, which may suggest a conserved role for the family of IQ‐motif‐containing proteins via calmodulin signaling and dendritic morphology. In summary, while elevated IQCK protein expression was observed in the AD models, a causal relationship between IQCK and the decline of dendritic spines in AD requires further studies.

## Author Contributions

**Juliet Akkaoui:** formal analysis (equal), methodology (equal), visualization (equal). **Dinesh Devadoss:** data curation (equal), formal analysis (equal), validation (equal). **Hongjie Wang:** data curation (equal), formal analysis (equal), investigation (equal), software (equal). **Alexandru Movila:** funding acquisition (equal), resources (equal), writing – original draft (equal). **Madepalli K. Lakshmana:** conceptualization (lead), formal analysis (equal), investigation (equal), methodology (equal), validation (equal), writing – original draft (equal).

## Conflicts of Interest

The authors declare no conflicts of interest.

## Data Availability

All the original data contributions presented in the study are included in the article and any further inquiries can be directed to the corresponding author.
